# A metaheuristic optimization-based approach for accurate prediction and classification of knee osteoarthritis

**DOI:** 10.1038/s41598-025-99460-4

**Published:** 2025-05-14

**Authors:** Amal G. Diab, El-Sayed M. El-Kenawy, Nihal F. F. Areed, Hanan M. Amer, Mervat El-Seddek

**Affiliations:** 1 Department of Communications and Electronics Engineering, MISR Higher Institute for Engineering and Technology, Mansoura, 35511 Egypt; 2 Department of Communications and Electronics Engineering, Delta Higher Institute of Engineering and Technology, Mansoura, 35111 Egypt; 3 Department of Electronics and Communications Engineering, Faculty of Engineering, Mansoura University, Mansoura, 35511 Egypt; 4 Department of Electronics and Communications Engineering, Horus University, New Damietta, 34517 Egypt; 5 Applied Science Research Center, Applied Science Private University, Amman, Jordan

**Keywords:** Knee osteoarthritis, Deep learning, Convolutional neural networks, Binary Greylag Goose optimizer, Data processing, Machine learning

## Abstract

Knee osteoarthritis (KOA) is a severe arthrodial joint condition with significant global socioeconomic consequences. Early recognition and treatment of KOA is critical for avoiding disease progression and developing effective treatment programs. The prevailing method for knee joint analysis involves manual diagnosis, segmentation, and annotation to diagnose osteoarthritis (OA) in clinical practice while being highly laborious and a susceptible variable among users. To address the constraints of this method, several deep learning techniques, particularly the deep convolutional neural networks (CNNs), were applied to increase the efficiency of the proposed workflow. The main objective of this study is to create advanced deep learning (DL) approaches for risk assessment to forecast the evolution of pain for people suffering from KOA or those at risk of developing it. The suggested methodology applies a collective transfer learning approach for extracting accurate deep features using four pre-trained models, VGG19, ResNet50, AlexNet, and GoogleNet, to extract features from KOA images. The numeral of extracted features was reduced for identifying the most appropriate feature attributes for the disease. The binary Greylag Goose (bGGO) optimizer was employed to perform this task, with an average fitness of 0.4137 and a best fitness of 0.3155. The chosen features were categorized utilizing both deep learning and machine learning approaches. Finally, a CNN hyper-parameter algorithm was performed utilizing GGO. The suggested model outperformed previous models with accuracy, sensitivity, and specificity of 0.988692, 0.980156, and 0.990089, respectively. A comprehensive statistical analysis test was performed to confirm the validity of our findings.

## Introduction

Osteoarthritis (OA) is one of the most frequent and debilitating chronic illnesses, accounting for the fourth major cause of disability worldwide^1^, with the knee being the most usually smitten joint. Pain is the defining sign of knee OA, driving patients to seek medical care and contributing to a lower quality of life^2^. Knee osteoarthritis (KOA) is a prevalent chronic ailment recognized as degenerative knee joint arthritis that results from 'wear and tear’ within the ligaments that connect the femur and tibial bone^3,4^.

Frequently, the disease is associated with gradual structural degradation of articular cartilage, causing patients to suffer permanent physical impairment. Knee OA has a significant global occurrence rate, as per the latest literature review on the epidemiology of OA^5^.

Older age, obesity^6^, and prior injury to the knee^7^ are all considered risk factors for OA, which results in pain that impairs function and lowers life’s quality. Total knee replacement (TKR), the definitive treatment for OA, is costly and has a short lifespan, particularly for those who are obese^8^. Consequently, early recognition of OA in the knee is essential for starting therapy, like losing weight and workouts, which effectively stop the evolution of OA in the knee and delay TKR^6,9^. Furthermore, several studies have emphasized the negative impact of knee osteoarthritis on the economy in terms of GDP loss^10^, direct healthcare cost burden^11^, and yearly productivity cost of employment loss^12,13^.

**Fig. 1:**
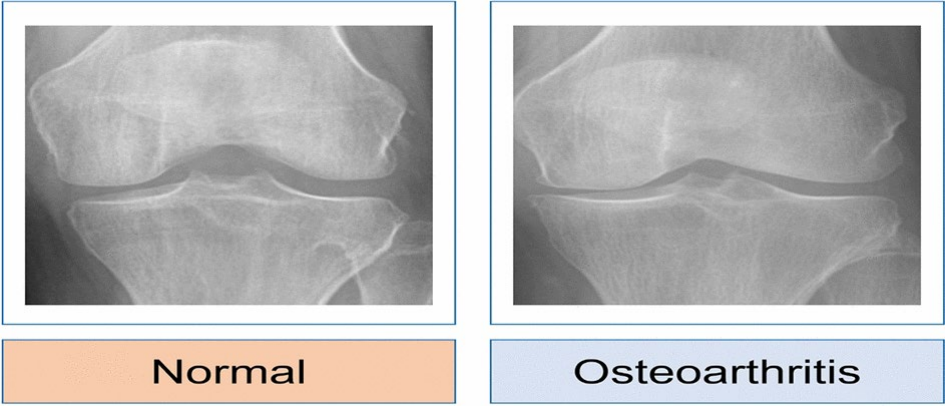
The normal knee joint and knee joint with osteoarthritis^27^.

KOA affects approximately one in every three individuals^14,15^. More than half of persons aged 65 and up have evidence of osteoarthritis, including that one joint. According to the World Health Organization’s (WHO) 2016 osteoarthritis report, 9.6% of men and 18.0% of women past the age of sixty had typical osteoarthritis. Among them, 80% have mobility issues, and 25% find it challenging to carry out their everyday duties^16^. According to the United Nations, 130 million people will suffer from KOA by 2050, with 40 million seriously crippled by the condition. KOA is one of the leading five factors that cause disability, posing a growing financial strain on society, mainly because of missed work hours and healthcare costs^17^. Figure 1 depicts the healthy knee joint and knee joint with osteoarthritis. Clinically, it is critical to diagnose this joint and determine the afflicted areas appropriately. X-ray, MRI, and CT modalities are utilized for scanning these areas to detect wear and tear, as well as other treatments like implanting and total knee replacement.

Radiography (X-ray) imaging is preferred for assessing OA^18^ because of its accessibility, cost-effectiveness, superior spatial resolution and contrast for tissues and bones. There are several forms of OA-related segmentation or categorization techniques to evaluate the knee that are broadly classed as classical approaches and deep learning (DL) approaches^19–21^. In current clinical procedures, OA intensity is typically assessed visually using radiography images, which are prone to inter-rater heterogeneity and time-consuming for big datasets^22^.

Deep learning (DL), a sophisticated form of artificial intelligence, is successfully used in various medical imaging tasks^23^. DL can potentially give a new technique for designing OA risk estimation algorithms that predict pain progression by extracting meaningful prognostic information from imaging scans in a timely and automated manner. CNN and other deep learning approaches automatically extract visual aspects from the model architecture through a sequence of transformations to enable the learning of complicated features^24,25^. CNN belongs to a deep learning technique that falls within the machine learning field of artificial intelligence (AI). CNNs are flexible, relatively simple, and slick for training, as a network learns during the tuning procedure using fewer parameters^26^. CNN’s overall design consists of a layer for input, hidden layers connected by a sequence of image filters, feed-forward network layers that show image filters on the input image, and an output layer wherein the feature is retrieved^20,25^. Integrating CNNs and transfer learning frameworks significantly improves the recognition of images for knee osteoarthritis.

This research intended to create and test algorithms for DL risk evaluation for forecasting the development of pain among individuals who have or are susceptible to osteoarthritis in the knee. DL approaches outperform conventional approaches based on clinical, demographic, and radiographic risk factors regarding pain progression prediction. In this paper, the images were processed, improved, and normalized. The suggested CNN and additional pre-trained algorithms were used for the feature extraction task, and a metaheuristic optimizer was employed to choose the best features among them. Lastly, apply the proposed deep neural network (DNN) architecture for categorizing these features.

The rest of the article is structured as follows: section "Related works" addresses recent research efforts in KOA diagnosis; Section "Material and methods" discusses the methodology for the suggested procedure and the feature selector models; Section "Evaluation criteria" illustrates the study’s significant findings; Section "Classification results and discussion" discusses the classification results and discussion; and section "Conclusion" discusses the study’s conclusion and suggestions.

## Research contribution

This work addresses the challenge of automatically classifying osteoarthritis in the knee using X-rays. This study presents the following key contributions:

1. A novel system is proposed to assist medical specialists in diagnosing KOA and classifying its severity as needed.
2. The classification models’ accuracy is boosted by implementing pre-processing methods that use a high pass filter to filter images in the frequency domain, highlighting the texture of trabecular bone and increasing classification accuracy.
3. The impact of the dataset’s imbalanced distribution is minimized, and a rebalancing process is also presented, dramatically increasing classification accuracy.
4. A DL model is proposed with the lowest misclassifications in the results.
5. The thoughtful CNN model is applied to extract the features out of the images in the dataset.
6. The significant features are selected by a bGGO optimizer.
7. The selected features are classified by K-nearest neighbor (K-NN), a decision tree (DT), a Multi-layer Perceptron (MLP) and a convolutional neural network (CNN) classifier.
8. A CNN hyper-parameter model is executed with a GGO.
9. A deep neural network (DNN) model is proposed for identifying the KOA features accurately.
10. The KOA recognition performance measures are evaluated against contemporary studies and pre-trained algorithms.

## Related works

Some studies have presented methods for classifying Knee Osteoarthritis utilizing various techniques, although the results are far from optimal. New OA classification algorithms are evolving as deep neural network topologies evolve.

In 2016, Antony et al.^28^ suggested an innovative technique that uses a deep convolutional neural network (DCNN) to categorize the intensity of OA in the knees from radiographs. The outcomes on X-ray images and KL grade dataset demonstrate a notable advancement over the state-of-the-art. In place of template matching, they suggested utilizing horizontal image gradients to train a linear SVM quicker and more precise than template matching. The resulting classification accuracy was 59.6%.

In 2017, Antony et al.^29^ presented a cutting-edge technique that automatically recognizes knee joints using a fully convolutional neural (FCN) network. By the weighted ratio optimization of two loss functions, namely category cross-entropy and mean-squared loss, they trained convolutional neural networks (CNNs) to evaluate the severity of knee osteoarthritis. They achieved a mean squared error of 0.898 and a multiple classes categorization accuracy of 60.3%.

In 2018, Tiulpin et al.^30^ suggested an innovative approach to identifying and classifying knee OA using standard radiographs. They used the deep Siamese network structure to classify OA. This architecture’s original purpose was to learn a similarity measure between image pairings. Two branches comprise the entire network, one for each input image. A probability distribution of grades across photos was utilized to assess the graded CAD system. They also tested a well-adjusted ResNet-34 network. The average multiclass accuracy was 66.71%.

In 2018, Suresha et al.^31^ trained a pre-trained networks (ImageNet) through a training approach alternating among object-categorization and region-proposal network fine-tuning, as shared feature across both was predicted to increase prediction reliability. Knee regions that were manually labeled served as ground truth for the region-proposal network’s training. The accuracy of their multiclass categorization was 88.2%.

In 2019, Abedin et al.^32^ employed Elastic Net (EN) and Random Forests (RF) to develop predicting approaches utilizing patient evaluation information and CNN trained only on an X-ray dataset. The within-subject association between the two knees was modeled using linear mixed-effects models (LMMs). The CNN, EN, and RF algorithms have root mean squared errors of 0.77, 0.97, and 0.94, respectively.

In 2019, Tiulpin et al.^33^ introduced an approach based on multimodal machine learning to forecast osteoarthritis progression that uses clinical examination findings, raw radiography data, and the patient’s previous health information. This approach was confirmed using an independent test collection of 3,918 knee pictures among 2129 participants. This approach produced an average precision (AP) of 0.68 (0.66–0.70) and an area under the ROC curve (AUC) of 0.79 (0.78–0.81).

In 2019, Chen et al.^34^ effectively deployed two deep convolutional neural networks for automated prediction of KOA and its degree of seriousness. The foundational X-ray scans for this approach were received from the OAI. The suggested method begins by recognizing the knee joints in the images utilizing a bespoke YOLOv2 network. They could categorize knee X-ray images into seriousness classifications utilizing the KL grading system after fine-tuning DenseNet, VGG, ResNet, and InceptionV3. Their knee joint identification approach had a recall of 92.2% and a mean Jaccard index of 0.858, while their calibrated VGG-19 model detected knee osteoarthritis severity with 69.7% accuracy.

In 2019, PU Patravali et al.^35^ developed an approach to calculate cartilage area/thickness utilizing several form descriptors. The generated descriptors achieved an accuracy of 99.81% for the KNN classifier and 95.09% for the DT classifier.

In 2019, PU Patravali et al.^36^ introduced an innovative method to investigate several segmentation strategies for the early identification of OA. The experiment employed various segmentation techniques, such as Sobel and Prewitt edge segmentation, Otsu’s method of segmentation, and texture-based segmentation. The various statistical features were calculated, analyzed, and categorized. The achieved accuracies were 91.16% for the Sobel approach, 96.80% for Otsu’s approach, 94.92% for the texture approach, and 97.55% for the Prewitt approach.

In 2020, Thomas et al.^37^ sought to develop an automated system for diagnosing the degree of severity of KOA using radiography. Despite using a large dataset, the approach’s effectiveness was assessed by contrasting its results to the opinions of radiologists specializing in musculoskeletal disorders. The radiograph images were enhanced automatically and then fed into a CNN model. They achieved an F1 score of 70% and overall accuracy of 71% over the whole tested dataset.

In 2020, Leung et al.^38^ introduced a KOA classification deep-learning algorithm built on sufferers’ knee images with complete knee replacement surgery. They contrasted it with individuals who didn’t have KOA. To discriminate between KL-based grade classes, a ResNet34 model with cross-validation was employed. The study employed a dataset of 4796 photographs obtained from the OAI. The model suggested has an accuracy rate of 72.7%. The restricted dataset size and transfer learning usage hampered the system’s ability to implement more accurately.

In 2021, Javed et al.^39^ evolved Resnet-14, a residual network that has been pre-trained, to forecast KL grades from radiograph data. A multicenter dataset has been employed to validate the network’s performance. The network obtained 98% accuracy and 98% AUC.

In 2021, Shivanand S. Gornale et al.^40^ proposed a novel method for detecting osteoarthritis by identifying the region of interest. A database of 1,173 knee X-rays was collected and manually graded by two independent medical specialists using the Kellgren and Lawrence grading system. The computation was accomplished using the histogram of the orientated gradient method and the local binary pattern (LBP). The calculated characteristics were categorized with a decision tree classifier. The proposed approach had an accuracy of 97.86% and 97.61%.

In 2022, Ribas et al.^41^ suggested an innovative technique for detecting early knee OA based on complicated network modeling and statistical data. The proposed network technique allowed for modeling the primary properties of the X-ray pictures while also increasing the separation between the control and OA groups. The suggested technique’s accuracy was 81.69%.

In 2022, Teo et al.^42^ introduced pre-trained InceptionV3 and DenseNet201 networks using the OAI dataset for extracting features from the OAI data set, which is divided into five categories based on osteoarthritis intensity. The SVM classifier is employed to categorize the features of the deep learning framework. The accuracy rate for DenseNet201-SVM is 71.33%.

In 2023, C. Guida et al.^53^ suggested a fusion approach that blends three distinct types: MRI, X-ray, and the patient’s clinical data into a single structure, increasing accuracy over the methods utilized independently. The fusion architecture was constructed utilizing two systems from previous studies trained using a limited dataset. It blended a conventional CNN for X-rays and a unique 3D MRI model. The study’s conclusions indicated that the utilized approach received performance accuracy ratings of 76%, which was inadequate and had to be improved.

In 2024, Anandh Sam Chandra Bose et al*.*^54^ utilized a CNN approach to extract characteristics by clinical imaging data. They utilized sophisticated approaches like PSO and Genetic Bee Colony (GBC) to uncover significant characteristics for improving ML models. Comparing approaches with optimized features to those trained with direct CNN features reveals significant accuracy, sensitivity, specificity, PPV, and NPV improvements across various ML techniques, such as SVM, KNN, RF, and Linear Discriminant Analysis (LDA). Features that GBC chose achieved 99.15% accuracy in binary categorization tasks. In multiclass classification, GBC characteristics paired with RF achieved an accuracy of 98.91%.

In 2024, Muhammed Yildirim and Hursit Mutlu^43^ created a hybrid model by extracting features utilizing Darknet53, Histogram of Directional Gradients (HOG), Local Binary Model (LBP), and Neighborhood Component Analysis (NCA). The dataset included 1650 knee images divided into five categories: standard, doubtful, mild, moderate, and severe—the experimental investigations compared the suggested method’s performance to eight distinct CNN Models. The developed model had an accuracy rating of 83.6%.

Lately, deep learning algorithms are being used in medical imaging to increase the precision of disease diagnosis. CNNs have been utilized in several research to classify knee osteoarthritis as either standard or osteoarthritis reliably.

The researchers succeeded in achieving satisfactory outcomes with a variety of approaches and materials. Every researcher aims to achieve the promised precision of X-ray image analysis for earlier KOA detection. Another thing to consider is that most current studies were conducted using osteoarthritis initiative (OAI) or MOST datasets, with an imbalanced data distribution. This study differs from earlier studies in that it used a variety of approaches and hybrid materials to achieve high accuracy, as well as an applied data-balanced strategy. Because it is challenging to categorize KOA images correctly, the obstacle was overcome by extracting characteristics from many deep neural models, selecting the best one, and then classifying them. Table 1 summarizes relevant studies concerning the diagnosis of knee osteoarthritis.

**Table 1:** An overview of relevant literature.

References	Method	Model	Dataset (Images)	Purpose	Accuracy
Antony et al.^28^	Applied a Convolutional Neural Network (CNN) for categorization utilizing: 1. A CNN pre-trained model to extract fixed features 2. Fine-tuning to the pre-trained CNN	-Linear SVM and sobel horizontal image gradients as the features -Imagenet -VGG16 -BVLC Caffenet -VGG-M-128	8892	Determined the degree of knee osteoarthritis automatically from radiographs by utilizing deep convolutional neural networks (DCNN)	-Fine-tuned network: 59.6% -Linear SVM: 94.2%(train) 95.2%(test)
Antony et al.^29^	Utilized a fully convolutional network (FCN) and a convolutional neural network (CNN) for simultaneous categorization and regression of localized knee images	CNN model (5 layers)	Two datasets: 1.Osteoarthritis Initiative (OAI) 3146 2.Multicenter Osteoarthritis Study (MOST) 1300	localized the knee joints automatically, then classified the images of the localized knee joints	-Detection: 100% 99.5% -Classification: 60.3%
Tiulpin et al.^30^	Utilized a Deep Siamese Convolutional Neural Network	fine-tuned resnet-34 network	5960 (OAI)	Used the Kellgren-Lawrence grading scale to automatically score the extent for knee OA	66.71%
Suresha et al.^31^	Used Deep learning approaches	-Imagenet (pre-trained model) -Fine-tune the regional proposal network	7549 (OAI)	- Detected the knee-region - Assessed the extent for knee osteoarthritis utilizing X-ray images	-Knee-region detection: 99.9% -Classification: 88.2%
Abedin et al.^32^	Used a Convolution Neural Network (CNN)	-Linear mixed effect models (LMM) , Elastic Net (EN) and Random Forests (RF) -Convolution neural network (CNN)	4,796	Predicted the level of KOA seriousness utilizing X-ray images only	Root mean square error for the CNN, EN, and RF frameworks equals 0.77, 0.97 and 0.94 respectively
Tiulpin et al.^33^	-Used Multi-modal machine learning-based model to predict OA progress -Used a Deep Convolutional Neural Network (DCNN)	Gradcam attention maps	8846	Predicted structural OA progression	-Area under ROC curve (AUC) = 0.79 (0.78–0.81) -Average Precision (AP) = 0.68 (0.66–0.70)
Chen et al.^34^	Implemented two deep convolutional neural networks (DCNN) to assess knee osteoarthritis’s seriousness (KOA)	-One-stage yolov2 algorithm -Fine-tuned editions of Densenet, Resnet, VGG, and Inceptionv3	N/A	Detected knee joints and classified a detected knee joint images using a unique alterable ordinal loss	69.7%
Thomas et al.^37^	Used Convolutional neural networks (CNNs)	Densenet	40,000 (OAI)	Developed an automated approach for assessing the seriousness of knee osteoarthritis using radiographs	71%
Leung et al.^38^	Used a deep learning framework	Resnet34 with cross-validation	4796 (OAI)	Classified knee osteoarthritis images	72.7%
Javed et al.^39^	Used a convolutional neural network (CNN) has six distinct directions utilizing class balance as well as data augmentation	Pre-trained residual network Resnet-14	917 (Clinical hospital center of Rijeka)	Detected anterior cruciate ligament damage at its early stage	98%
Ribas et al.^41^	Used a complex theory of networks for extracting textural features	-Support Vector Machine (SVM) -K-Nearest Neighbors (k-NN) -Linear Discriminant Analysis (LDA)	688 (OAI)	Presented a novel technique based on complex theory of networks concepts for extracting textural information linked to OA from radiographic knee X-ray images for early knee OA detection	81.69%
Teo et al.^42^	Used a deep DL model	-Pre-trained inceptionv3 and densenet201 frameworks -SVM model	1,000 (OAI)	Extracted the features from OAI dataset and then classified OAI images	71.33%
Guida et al.^53^	Used a deep DL model	CNN		To classify OA severity	76%
Anandh Sam Chandra Bose et al*.*^54^	Used a deep DL model	-Ensemble TL-ACO -Alex-Net -custom Isr-Net -k-means clustering based on PCA -ACO optimizer	OAI	To grade KOA	SVM: 89.89% KNN: 85.44%
Muhammed Yildirim and Hursit Mutlu^43^	Applied a DL model	-HOG, LBP, and NCA models	1650	Classified the KOA images	83.6%

## Material and methods

**Fig. 2:**
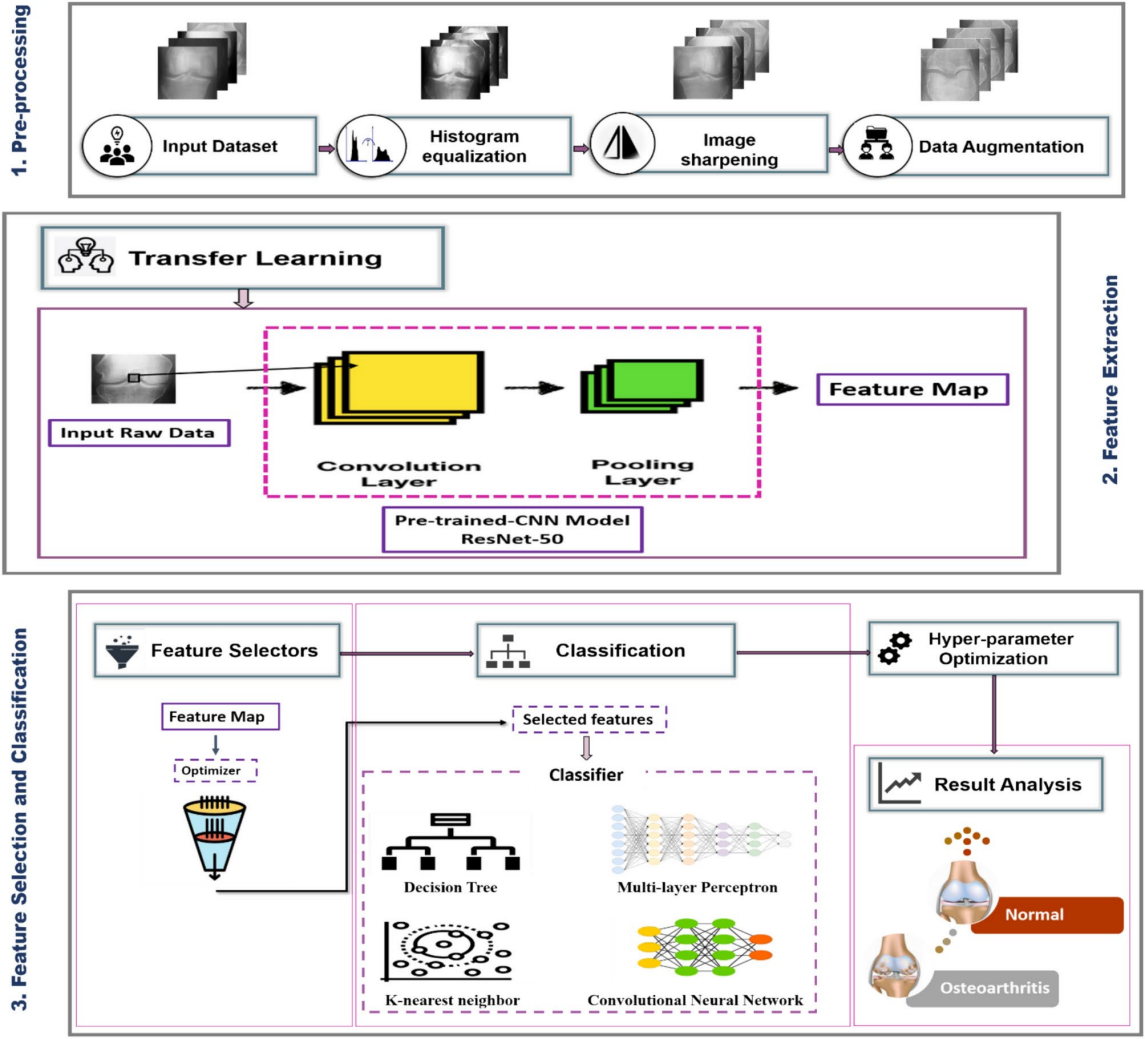
The general scheme for the suggested framework.

The steps involved in the proposed classification approach for knee OA diagnosis in this study are the gathering and preparation of data, the extraction and selection of features, and the recognition of image labels; this is illustrated in Fig. 2. A dataset of knee x-ray images has been downloaded. The gathered dataset was then subjected to the preprocessing procedures. Image enhancement techniques include frequency-domain filtering, histogram equalization, and sharpening. After the dataset has been collected and preprocessed, four common deep-learning approaches, AlexNet^44,45^, VGG19^46^, ResNet-50^47,48^, and GoogleNet^49,50^, were trained, evaluated, and contrasted with choosing the most effective one for detecting KOA instances. The chosen model received the processed images, and during training, their parameters were adjusted to improve accuracy. Then, features are extracted from the input images by the highest-performing model. The optimal feature collection is then found by processing the retrieved features using the suggested feature selection procedure. An optimized CNN classifier is trained to utilize the optimal set of features to determine the case of the input image. The following subsections will thoroughly explain the suggested framework’s methodology utilizing the KOA dataset.

### Dataset description

The knee osteoarthritis graded data set provided knee X-ray images utilized in this study to train the proposed framework. The images are accessible on Kaggle^27^ and collected through the Osteoarthritis Initiative (OAI). There are a total of 3835 knee images, separated by two grades. In the dataset, all images are carefully assessed by competent clinicians as usual or osteoarthritis, with the distribution of each grade displayed in Table 2. The dataset’s images were scaled down to 224 × 224 pixels for easier processing by the model due to their uniform size.

**Table 2:** Distribution of knee osteoarthritis dataset.

Dataset split-up	Grade	Number of images	% of total
Train	Normal	810	21.12%
	Osteoarthritis	1540	40.15%
Validation	Normal	210	5.47%
	Osteoarthritis	430	11.21%
Test	Normal	569	14.83%
	Osteoarthritis	276	7.19%

### Data preparation

The data Preparation step is essential in analyzing images due to increased standards for high-quality data and consistency.

i. Data Augmentation and Balancing.

**Fig. 3:**
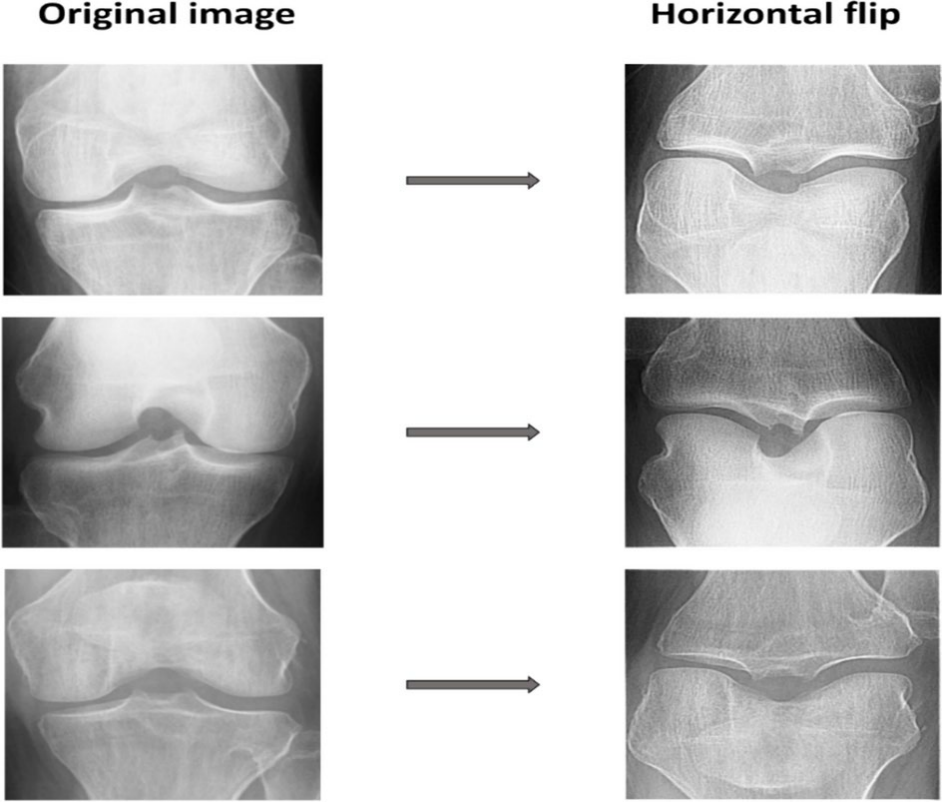
The implemented operation of data augmentation technique.

Figure 3 depicts the implementation of data enhancement methods on images to boost the dataset’s size while preventing overfitting. The expanded data set enhanced the model’s reliability and accuracy. The flipping approach was used on the dataset. It is possible to build a significantly more extensive and diverse dataset to train the deep learning algorithm using the data augmentation method, making it possible to create additional images with minimum changes to the original ones. When these methods are applied, a model can comprehend the core characteristics of the images since it is exposed to a broader range of permutations. After data augmentation, the dataset contains 5132 images. Table 3 shows the ultimate dataset utilized to train the network for this investigation, and Table 4 summarizes the distribution of all datasets. These knee joints are categorized as train, validation, and test datasets.

**Table 3:** Distribution of the Rebalanced Dataset.

Dataset split-up	Grade	Number of images	% of total
Training set	Normal	1620	31.56%
	Osteoarthritis	1540	30.00%
Validation set	Normal	420	8.18%
	Osteoarthritis	430	8.37%
Testing set	Normal	569	11.08%
	Osteoarthritis	552	10.75%

**Table 4:** Distribution of all dataset.

Dataset	No. of images before augmentation	No. of images after augmentation	% of total
Normal	1589	2609	50.83%
Osteoarthritis	2246	2523	49.16%

The dataset has a highly uneven distribution; the standard class data is significantly less than the osteoarthritis class in the sets for training and validation, as the osteoarthritis class data is much less than the regular class in the testing set. To avert biasing the training results, the suggested framework attempts to ensure data balance by randomly choosing an equal number of images for every category. This process is known as “data balancing” Ideally, each class should have a detection rate that is nearly or the same. With a balanced dataset, a model can achieve higher detection rates, accuracy, and precision, as demonstrated in the abovementioned examples. To reduce the negative impact on the results., the flipping technique artificially rebalanced the dataset.

ii. Data Pre‐processing.

**Fig. 4:**
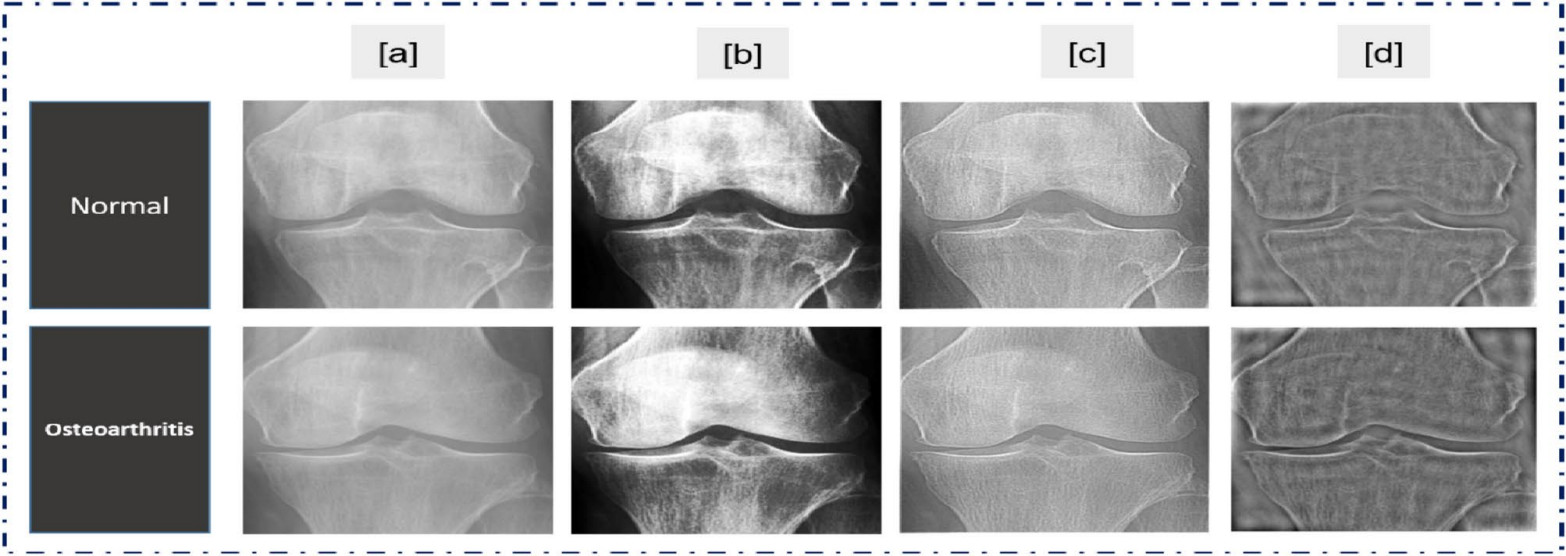
Image pre‐processing process. (**a**) The input images. (**b**) The pre-processed images by histogram equalization. (**c**) The pre-processed images by sharpening filter. (**d**) The pre-processed images by a frequency domain high‐pass filter.

The frequency domain filter is applied to the images first, and the histogram is normalized to enhance the features of trabecular bone texture and improve recognition accuracy. Second, image sharpening is utilized in a customizable function to reduce noise and equalize histograms. Figure 4 depicts the workflow for the three primary processes: histogram normalization, frequency-domain filtering, and image sharpening.

**Fig. 5:**
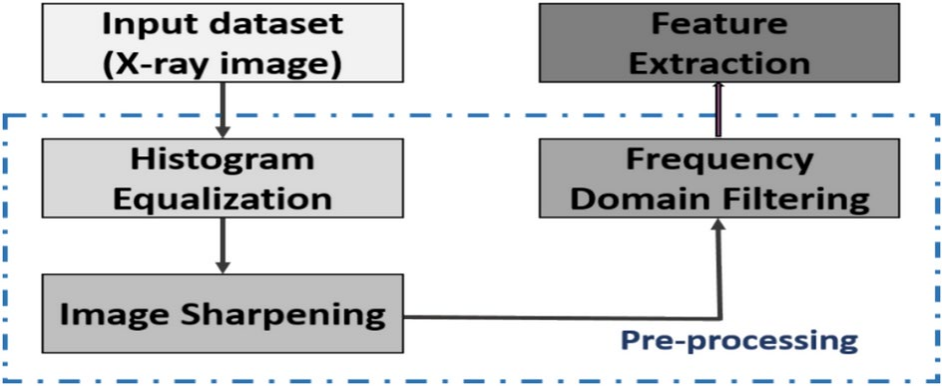
Image pre**‐**processing steps.

The non-linear histogram normalization technique improves the filtered image’s contrast since most X-ray images in the OAI dataset have poor contrast. The image’s intensity is then returned^52^. Figure 5 depicts the pre-processing findings of the photographs. Equation (1) illustrates the formula for performing histogram equalization on the images, where 'r' represents the input pixel’s value and 's' represents the output pixel’s value. 'L' denoted the image’s highest pixel values. Equation (2) expresses the likelihood of r_j_ intensity level occurrence, where n_j_ is the numeral of pixels with r_j_ intensity and ‘MN’ is the whole numeral of the image’s pixels.

1 $${\text{sk}} = {\text{T}}\left( {{\text{rk}}} \right) = \left( {{\text{L}} - 1} \right) \mathop \sum \limits_{j = 0 }^{k} p_{r} (r_{j} )$$

2 $${\text{p}}_{r}{(r}_{j})=\frac{{\text{n}}_{j}}{MN}$$

### Feature extraction

The main pre-trained models used for feature extraction were Alex-Net, Google-Net, VGG-Net, and ResNet50. Those models include layers that incorporate both linear and nonlinear processes that were learned in a combined manner. Extracting features from deep learning frameworks, such as ResNet50, can be divided into multiple steps. The data was fed into the ResNet50 model, and backpropagation was utilized to train the network, adjusting the neurons’ weights and biases to reduce the loss function. Here, features were extracted from images with pinpoint accuracy due to the strength and efficacy of deep learning algorithms like ResNet50.

### Feature selection

The X-ray image features are reduced by using the feature selection technique. Increased correlation among characteristics improves the accuracy of classification. This study applies a Greylag Goose (GGO) optimizer to perform the feature selection task.

### GGO algorithm

The Greylag Goose Optimization (GGO) algorithm is used for optimization in the present study. There are many advantages to the GGO optimizer, including the colony functions independently of any higher authority (Modularity), the task is completed effectively generally even if multiple agents fail (Robust), and network adjustments can spread quickly (Speed). However, it is difficult to predict behavior based solely on the rules themselves (behavior); it is impossible to understand how a colony functions without knowing how an agent functions (knowledge), and any departure from these fundamental norms changes the collective behavior (sensitivity). The GGO algorithm starts by creating a random population of individuals, each representing a potential fix for the problem. This population called a gaggle, has size n and is represented by the symbol X_i_ (i = 1, 2, …, n). Any individual is assessed using an objective function, F_n_, of choice. The best solution, or leader, Xi, is found by computing the objective function of every individual (agent) and is indicated by P. Next, the population is dynamically divided into two categories through the GGO algorithm: an exploration group (n_1_) and an exploitation group (n_2_). Considering the best solution obtained, each iteration has different solutions in every set. 50% of the population is initially split equally between exploration and exploitation groups via the GGO algorithm. The numeral of agents in the exploitation group (n_2_) rises while the numeral of agents within the exploration group (n_1_) falls as the iterations continue. After three successive iterations, the optimal solution’s objective function value remains constant. The algorithm raises the number of agents within the exploration group to find a better solution and stay away from local optima in that scenario (n_1_).

#### Exploration operation

Exploration is responsible for finding intriguing sections of the search space and preventing local optimum stagnation by moving toward the optimal answer. Moving towards the best solution: using this strategy, the geese explorer will look for intriguing new places to explore near its present position. The exploration is performed by continually evaluating several potential neighboring possibilities to determine the most excellent fitness. For the A and C vectors adjusted as A = 2a.*r*_1_ − a and C = 2.*r*_2_ throughout iterations with the parameter altered linearly from 2 to 0. The GGO algorithm employs the formulae that follow to do this:

3 $${\mathbf{X}}\;(t + 1) = {\mathbf{X}}*\left( t \right) - {\mathbf{A}}.\left| {{\mathbf{C}}.{\mathbf{X}}*\left( t \right) - {\mathbf{X}}\left( t \right)} \right|$$

where (*t*) is an agent at iteration *t*. The ^∗^ (*t*) is the optimal solution (leader) position. The updated position of the agent is (*t* + 1). The *r*_1_ and *r*_2_ values change arbitrarily within the range of [0,1]. The formula that follows is utilized to assist in choosing three random search agents (paddlings), termed _*Paddle*1_, X_*Paddle*2_, and X_*Paddle*3_, to push agents not to be affected by one leader position to gain greater exploration. The current search agent’s location will be adjusted to correspond for ||≥ 1.

4 $${\mathbf{X}}(t + { 1}) \, = w_{{1}} * {\mathbf{X}}_{paddle\;1} + {\mathbf{z}} * w_{{2}} * ({\mathbf{X}}_{{paddle\;{2}}} - {\mathbf{X}}_{{paddle\;{3}}} ) \, + \, ({1 } - {\mathbf{z}}) * w_{{3}} * ({\mathbf{X}} - {\mathbf{X}}_{paddle\;1} )$$

where [0, 2] is where the values of *w*_1_, *w*_2_, and *w*_3_ are updated. The formula that follows is used to calculate the parameter z, which is decreasing exponentially.

5 $${\text{z }} = { 1 } - \, (t/t_{\max } )^{{2}}$$

where *t* is the iteration numeral and *tmax* is the maximum numeral of iterations. For *r*_3_ ≥ 0.5, the second updating procedure, in which the values of the a and A vectors are reduced, is as follows.

6 $${\mathbf{X}}(t + { 1}) \, = w_{{4}} * |{\mathbf{X}}^{ * } (t) \, - {\mathbf{X}}(t)|.e^{bl} .{\text{Cos}} ({2}\pi l) \, + \, [{2}w_{{1}} (r_{{4}} + r_{{5}} )] * {\mathbf{X}}^{ * } (t)$$

where *l* is a random value in [− 1, 1] and *b* is a constant. While *r*_4_ and *r*_5_ are updating in [0, 1], the *w*_4_ parameter is updating in [0, 2].

#### Exploitation operation

The task of enhancing the current solutions falls to the exploitation team. At the end of each cycle, the GGO determines who is the most fit and gives them the appropriate prize. The GGO uses two distinct tactics to accomplish its exploitation goal, which are explained below. Moving in the direction of the best solution: The optimal solution is reached by using the subsequent formula. The three solutions (sentries), X_*Sentry*1_, X_*Sentry*2_, and X_*Sentry*3_, direct other individuals (X_*NonSenttry*_) to adjust their positions in anticipation of the prey’s predicted position. The subsequent formulas illustrate the position update procedure.

7 $$\begin{gathered} {\mathbf{X}}_{1} = {\mathbf{X}}_{senstry\;1} - {\mathbf{A}}_{1} .|{\mathbf{C}}_{1} .{\mathbf{X}}_{senstry\;1} - {\mathbf{X}}| \hfill \\ {\mathbf{X}}_{2} = {\mathbf{X}}_{senstry\;2} - {\mathbf{A}}_{2} .|{\mathbf{C}}{1}.{\mathbf{X}}_{senstry\;2} - {\mathbf{X}}| \hfill \\ {\mathbf{X}}_{3} = {\mathbf{X}}_{senstry\;3} - {\mathbf{A}}_{3} .|{\mathbf{C}}{1}.{\mathbf{X}}_{senstry\;3} - {\mathbf{X}}| \hfill \\ \end{gathered}$$

where A = 2a is used to derive A_1_, A_2_, and A_3_. C = 2*r*_2_ is used to determine *r*_1_ − a and C_1_, C_2_, and C_3_.

#### Searching the area around the optimal solution

When flying, the most promising option is situated near the best answer (leader). This leads certain individuals to look for improvements by exploring areas near the optimal response, called X_*Flock*1_. The following equation is used by the GGO to carry out the previously indicated procedure.

8 $${\mathbf{X}}(t\, + \,{1})\, = \,{\mathbf{X}}(t)\, + \,{\mathbf{D}}({1}\, + \,{\mathbf{z}})\, * \,w\, * \,({\mathbf{X}}\, - \,{\mathbf{X}}_{Flock\;1} )$$

#### Selection of the best solution

**Fig. 6:**
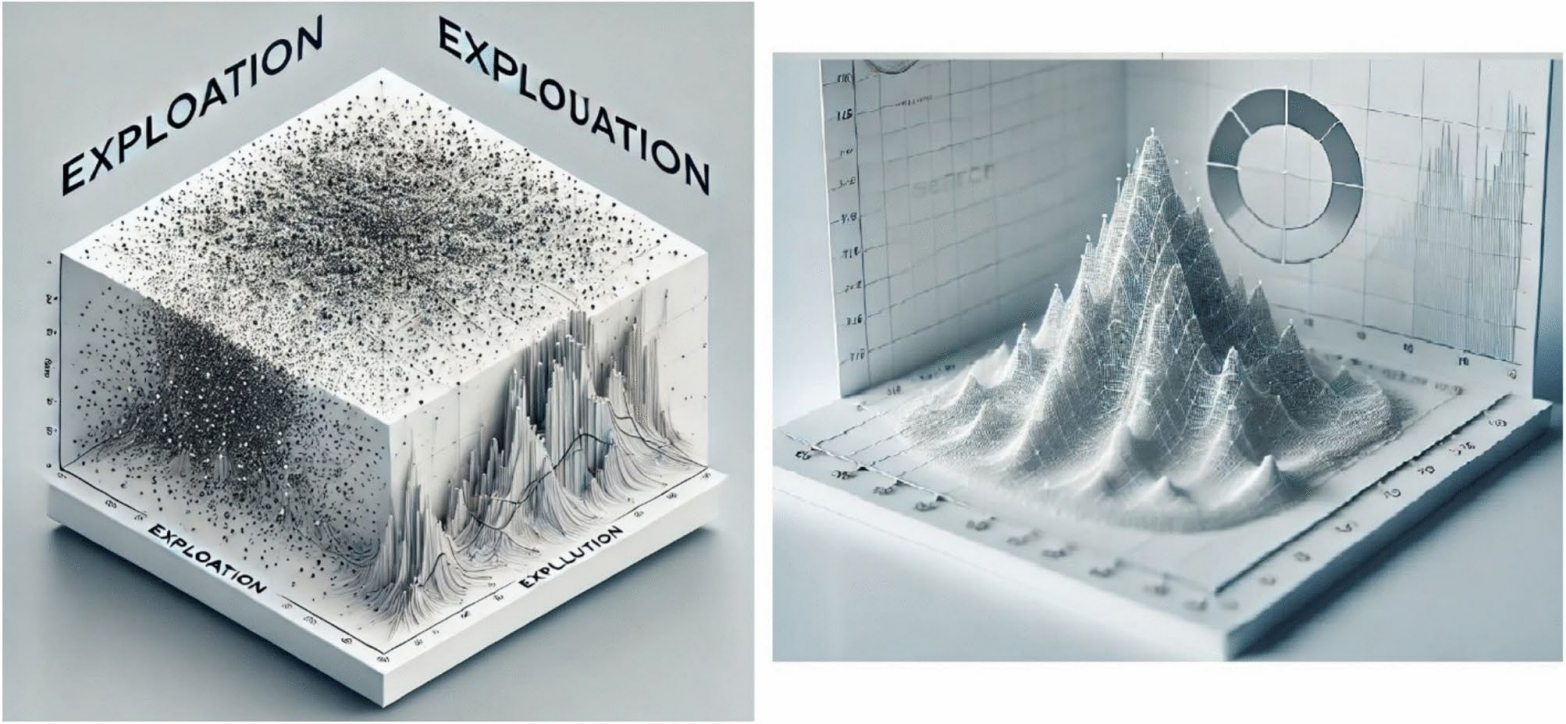
Algorithm’s steps: exploration, exploitation, and dynamic groups.

The GGO has outstanding exploration capabilities since it utilizes a mutation approach and scans members within the exploration category. The GGO’s powerful exploring capability allow it to defer convergence. The GGO pseudo-code is observable and can be found in algorithm 1. We first supply population size, mutation rate, and number of iterations to GGO. The GGO then divides the participants to two groups: those that engage in exploitative labor and those who engage in exploratory work. Throughout the iterative process of identifying the optimal solution, the GGO approach adjusts each group’s size dynamically. Every team uses two methods to complete its duties. The GGO arbitrarily rearranges the responses among iterations to offer diversity and in-depth study. A component of the solution from exploration group may move to exploitation group in a single iteration as seen below. The GGO’s elitism method ensures that the leader remains in place along the operation. Figure 6 depicts each stage of the GGO algorithm utilized to update the locations to the exploration group (*n*_1_) and exploitation group (*n*_2_). The parameter *r*_1_ is adjusted throughout iterations, as expressed in Eq. (9).

9 $${\text{r}}_{1} = {\text{c }}\left( {1 - {\raise0.7ex\hbox{${\text{t}}$} \!\mathord{\left/ {\vphantom {{\text{t}} {t_{max} { }}}}\right.\kern-0pt} \!\lower0.7ex\hbox{${t_{max} { }}$}}} \right)$$

where *c* represents a constant, *t* denotes the current iteration, and *t*_*max*_ represents the number of iterations. GGO updates the agents in the search space at the end of each iteration, and their positions in the exploration and exploitation groups are switched around as random. GGO gives back the optimal solution in the last stage.

### Binary GGO algorithm

Feature selection is one of the most important steps in analyzing data, as feature selection aims to minimize the data’s high dimensionality by removing irrelevant or redundant information. They have, therefore, been applied in a range of fields as the fundamental goal of this feature selection optimization technique is to identify important characteristics that minimize classification errors. A minimized optimization problem is a mathematical description of feature selection. The GGO algorithm’s results will be solely binary, with values of 0 or 1, If there are any issues with feature selection. To facilitate the process of selecting features within the dataset, the suggested GGO method’s continuous values will be transformed into binary values [0, 1], as shown in the phases of Algorithm 2.

**Figure:**
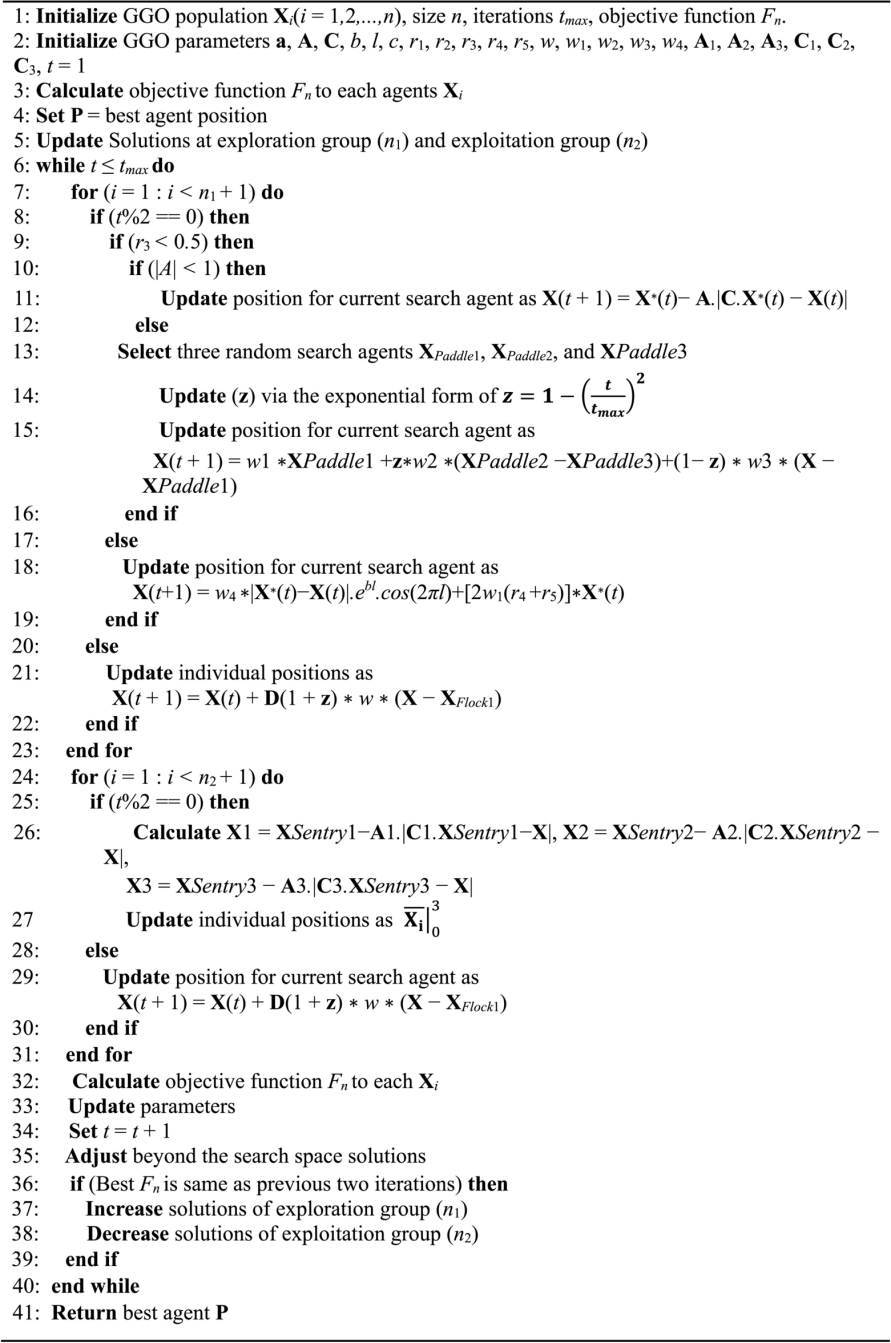
Algorithm 1: GGO Algorithm

**Figure:**
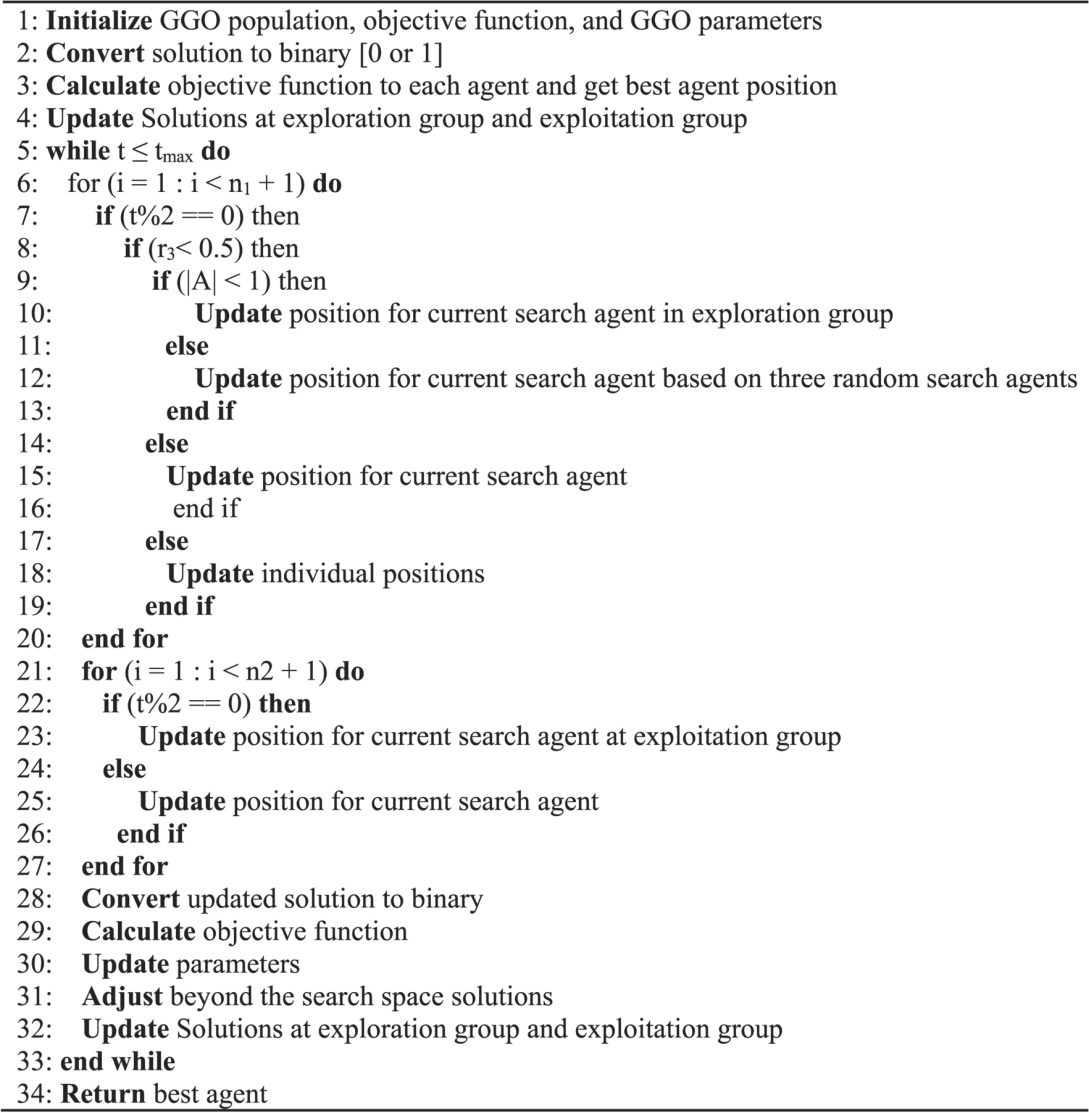
Algorithm 2: bGGO Algorithm

The Eq. (10) used in this study is based on the *Sigmoid* function and is represented as follows:

10 $$\begin{gathered} {\text{x}}_{{\text{ d }}}^{{{\text{t}} + 1{ }}} = \left\{ {\begin{array}{*{20}c} {1 if Sigmoid\left( m \right) \ge 0.5} \\ {0 otherwise, } \\ \end{array} } \right. \hfill \\ {\text{Sigmoid }}\left( {\text{m}} \right) = \frac{1}{{1 + {\varvec{e}}^{{ - 10\left( {{\varvec{m}} - 0.5} \right)}} }} \hfill \\ \end{gathered}$$

where ${\text{x}}_{\text{ d }}^{\text{t}+1}$ denoted the binary solution at iteration *t* and dimension *d*. The *Sigmoid* function is scaling the resultant solutions to binary ones. The value will vary to 1 if *Sigmoid*(*m*) exceeds 0.5. Alternatively, it will stay 0. The *m* parameter reflects the features selected by the algorithm.

Algorithm 2 provides a full explanation of the binary GGO method. The GGO algorithm has a computing complexity of *O* (*t*_*max*_ × *n*) and will be *O* (*t*_*max*_ × *n* × *d*) for the *d* dimension. The binary GGO algorithm uses the objective equation F_n_ to evaluate the quality of a solution. The following Eq. (11) formula represents the classifier’s error rate, *E*_*rr*_, using F_n_.

11 $$F_{n} = \alpha Err + \beta \frac{\left| s \right| }{{\left| S \right|}}$$

where *s* denotes a set of the selected feature, while *S* rep\/ + resents a set of missing features, β = 1—α and α ∈ [0, 1] indicates the population relevance of the specified trait. The strategy is successful if it can offer a subset of features with a minimal rate of errors in categorization. The only factor in classifier selection is the shortest path between the training and query instances.

### Image classification

Finally, a categorization approach is applied to the extracted and refined collection of features. Machine learning and deep learning classifiers are utilized for sorting KOA images, specifically, convolutional neural network (CNN), decision tree (DT), K-nearest neighbor (K-NN), and multi-layer perceptron (MLP) classifiers.

## Evaluation criteria

### Performance metrics to the pre-trained model and classifier

Using the confusion matrix, measurements like accuracy, precision, recall, and F1-score can be calculated by comparing expected labels against true ones. The confusion matrix consists of four categories: True Positive (TP) value, True Negative (TN) value, False Positive (FP) value, and False Negative (FN) value. When the actual and anticipated classes are knee OA, a TP accurately forecasted and signified the case. TN relates to situations where the actual and projected classes do not include knee OA. FP occurs when the anticipated class is knee osteoarthritis. However, the actual class is different. FN is cases where knee OA is the actual class but the projected class differs. The most reliable method for detecting and classifying cases of osteoarthritis in the knee was determined to be the model that performs the best. Table 5 describes the evaluation metrics.

**Table 5:** The description of the utilized evaluation metrics.

Metric	Abbreviations & formula	Explanation
Accuracy (ACC)	ACC = $\frac{TP+TN}{ TP+FN+TN+FP}$	Measures how frequently a model creates correct predictions compared to how often it makes wrong predictions
Sensitivity (SENS)	SENS = $\frac{TP}{TP+FN}$	Describes the rate with which a model predicts future occurrences of KOA
Specificity (SPEC)	SPEC = $\frac{TP}{TP+FN}$	Evaluate the system’s ability to predict adverse outcomes accurately
Positive predictive value (P-value)	P-Value = $\frac{TP}{TP+FP}$	Defines as the ratio of accurate optimistic KOA forecasts to total predictions of KOA. This metric compares the number of accurately categorized true positives to all positive samples
Negative predictive value (N-value)	N-Value = $\frac{TN}{TN+FN}$	Measures the opposite percentage
F1-score	F1-score = $\frac{2TP}{2TP+FP+FN}$	F-Measure combines accuracy and sensitivity measures using the harmonic mean

### Performance metrics to the optimizers

The following metrics are used in experiments to assess how well the suggested algorithm selects features for assessment (see Table 6). If *M* denotes the number of repetitions, *g* ∗ denotes the best solution, and *N* indicates the overall numeral of points, the ideal answer is *g* ∗ . *L* represents a point’s class, *C* represents the classifier’s output, and *M atc*ℎ indicates the degree of matching between the two inputs. ${g}_{j}^{*}$ denotes vector size, while *D* represents dataset size.

**Table 6:** The evaluation metrics used in experiments to evaluate how well the suggested optimizers select features for assessment.

Metric	Formula	Explanation
Average Error	Avg. Error = 1—$\frac{1}{M}$ $\sum_{j=1}^{M}\frac{1}{N} \sum_{i=1}^{N}\text{M atch}(\text{Ci},\text{ Li})$	Indicates the average number of errors caused by the characteristics selected to include in the subset. This statistic is crucial since it provides an approximate estimate of how the categorizing system performs based on the selected subset of features
Average Select-Size	Avg. Select Size $= \frac{1}{M} \sum_{j=1}^{M}\frac{\text{Size}({g}_{j}^{*})}{D}$	Indicates the standard numeral of features that the algorithm selects to optimize. This statistic can provide an approximation of the complexity of the categorizing system and the numeral of features necessary for satisfactory performance, making it a relevant quality indicator
Best Fitness	Best Fitness = ${Min}_{j=1}^{M}{g}_{j}^{*}$	Represents the maximum fitness value achieved by the optimized feature subset. It is significant since it demonstrates the categorization efficiency achievable with the given feature subset
Worst Fitness	Worst Fitness = ${Max}_{j=1}^{M}{g}_{j}^{*}$	Represents the least fitness values attained by the selected subset of features throughout the optimization. This number indicates the lowest potential categorization performance that may be obtained with the specified feature subset, making it critically important
Standard Deviation (SD)	SD = $\sqrt{\frac{1}{M-1} \sum {({g}_{j}^{*}-Mean)}^{2}}$	Determines the probable results obtained from the selected feature subsets during optimization. It is significant since it gives information on the dependability of the optimization technique and the prioritized feature set’s potency
Mean	Mean = $\frac{1}{M} \sum_{j=1}^{M}{g}_{j}^{*}$	Describes a distribution’s center or “typical” value. It is referred to as a location measure since it indicates where the central features are

### Feature extraction results

Metrics including F1-score, N-Value, P-Value, sensitivity, and accuracy are employed to assess extracted features’ efficacy. Suppose the extracted features exhibit superior precision, sensitivity, specificity, F1-score, and a low P-value. In that case, the extraction method succeeded in identifying the most significant features of the categorization task (see Table 7). The study’s feature extraction technique used the ResNet-50 deep learning model, which produced an accuracy of 88.60%. The findings shown in the table show that the feature retrieved using ResNet-50 outperforms other deep neural networks. As a result, the suggested methodology’s subsequent phases use such a network. This level of performance indicates that ResNet-50 can ideally select and include the most valuable features from the provided dataset, which is an essential capacity for addressing the image classification problem.

**Table 7:** Assessing the characteristics that were derived with CNN deep neural networks.

Model	Accuracy	Sensitivity (TRP)	Specificity (TNP)	P-Value (PPV)	N-Value (NPV)	F1-Score
GoogleNet	83.60%	84.30%	83%	83.70%	83.60%	0.839
ResNet-50	**88.60%**	**97.20%**	78.90%	83.90%	**96.10%**	**0.9005**
AlexNet	83.10%	78.40%	87.80%	87%	79.70%	0.8247
Vgg-19	87.52%	86.80%	**88.28%**	**89.35%**	85.55%	0.8808

This method’s feature extraction of ResNet-50 suggests that further optimization and extension into other domains could yield substantially greater success in the future. As a performance indicator, this shows how deep learning technology is developing and how well-suited it is to handle different challenging issues. Thus, future directions for technological progress in machine learning and artificial intelligence require that models like ResNet-50 be essential for obtaining improved outcomes across various domains.

### Feature selection results

Feature selection strategies are employed to refine the gathered features after the feature extraction procedure. A range of metrics is employed to assess the selected features’ effectiveness, including best-fitness, worst-fitness, average error, average fitness, average select size, and standard deviation fitness. When evaluating the outcomes of selected features, best fitness, worst fitness, average error, average fitness, average select size, and standard deviation fitness can be used to gauge quality, complexity, stability, robustness, and possession of insightful information on the classification technique’s efficiency. The results of the criteria for evaluation rely on the suggested feature selection strategy are shown in Table 8, along with a comparison to the other approaches: binary Greylag Goose Optimization (bGGO), binary Firefly Algorithm (bFA), binary Satin Bowerbird Optimizer (bSBO), binary Grey Wolf Optimization (bGWO), binary Particle Swarm Optimization (bPSO), binary Bat Algorithm (bBA), binary Genetic Algorithm (bGA), binary Multi-verse Optimization (bMVO), and binary Whale Optimization Algorithm (bWOA). It is evident from the outcomes obtained that the suggested feature selection strategy is superior to any feature selection techniques found in related works. The outcomes demonstrate the superior performance and efficacy of the suggested approach for identifying the necessary feature set required to categorize KOA cases.

**Table 8:** Evaluation of the suggested optimization algorithm against alternative optimization algorithms for the chosen set of features.

Metric	bGGO	bGWO	bPSO	bBA	bGA	bMVO	bSBO	bFA	bWOA
Avg. Error	**0.2905**	0.3677	0.4015	0.4111	0.4013	0.3782	0.4098	0.3999	0.3813
Avg. Select-Size	**0.3033**	0.5033	0.5033	0.6427	0.6667	0.5998	0.6736	0.5378	0.4457
Avg. Fitness	**0.4137**	0.4299	0.4283	0.4512	0.4361	0.4580	0.4680	0.4802	0.4413
Best Fitness	**0.3155**	0.3502	0.4086	0.3409	0.4002	0.3832	0.4111	0.3989	0.3446
Worst Fitness	**0.4140**	0.4171	0.4763	0.4425	0.4763	0.5012	0.4908	0.4965	0.4597
Standard deviation-Fitness	**0.2360**	0.2407	0.2401	0.2500	0.2423	0.2908	0.3010	0.2769	0.2423

## Classification results and discussion

Various classifiers are utilized in this study, such as K-nearest neighbor (K-NN), a decision tree (DT), Multi-layer Perceptron (MLP) and convolutional neural network (CNN) classifiers. Several metrics, including time, F1-score, N-value, P-value, sensitivity, and specificity, can be employed to evaluate the effectiveness of the optimized classifiers. According to these measures, if the selected features are perceptive and can reliably differentiate between the different KOA picture classes, then the optimized classifiers can achieve high classification performance. The categorization results before and after selecting a feature are shown in Table 9. This table makes it clear that the classification outcomes with the suggested feature selection outperform the classification with the previous feature selection.

**Table 9:** The classification outcomes that were attained both using and without using the suggested feature selection technique.

	Accuracy	Sensitivity (TRP)	Specificity (TNP)	P-Value (PPV)	N-Value (NPV)	F1-Score
Before Feature Selection	0.8860	0.9720	0.7890	0.8390	0.9610	0.9005
After Feature Selection	**0.988692**	**0.980156**	**0.990089**	**0.999822**	**0.965050**	**0.999989**

First, convolutional neural networks (CNN) classifiers are used. Table 10 shows the results obtained utilizing the suggested strategy and alternative ways of optimizing CNN using various optimizers. The GGO-CNN model outperformed other cutting-edge classifier models built with the CNN approach, as evidenced by its accuracy of 0.988692. With a 0.974479 accuracy, the GWO-CNN-based approach yielded the second-best classification results. It was followed with PSO-CNN-based approach, which scored 0.969067; the WOA-CNN-based model, which achieved a score 0.96545; and the BBO-CNN-based approach, which produced the least accurate outcomes, with a 0.9425 accuracy.

**Table 10:** The Classification outcomes for various optimization algorithms based on CNN.

Model	Accuracy	Sensitivity (TRP)	Specificity (TNP)	P-Value (PPV)	N-Value (NPV)	F1-Score	Time(s)
GGO-CNN	**0.988692**	**0.980156**	**0.990089**	**0.999822**	**0.965050**	**0.999989**	**110.7436**
GWO-CNN	0.974479	0.987518	0.900202	0.983643	0.958176	0.985577	130.7436
PSO-CNN	0.969067	0.984126	0.900202	0.979320	0.948471	0.981717	137.7436
WOA-CNN	0.96545	0.981717	0.900202	0.976255	0.948040	0.978979	141.7436
BBO-CNN	0.959409	0.976935	0.910416	0.969067	0.941558	0.972985	145.7136

**Fig. 7:**
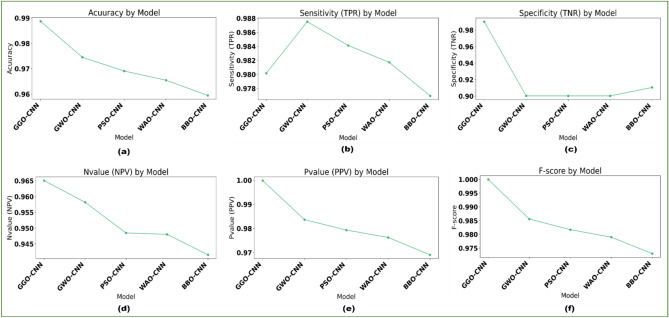
The results obtained by the CNN-based classifier as compared to the other optimization techniques when optimized with the proposed bGGO algorithm (**a**) Accuracy, (**b**) Sensitivity, (**c**) Specificity, (**d**) N-value, (**e**) P-value, and (**f**) F1-score.

**Fig. 8:**
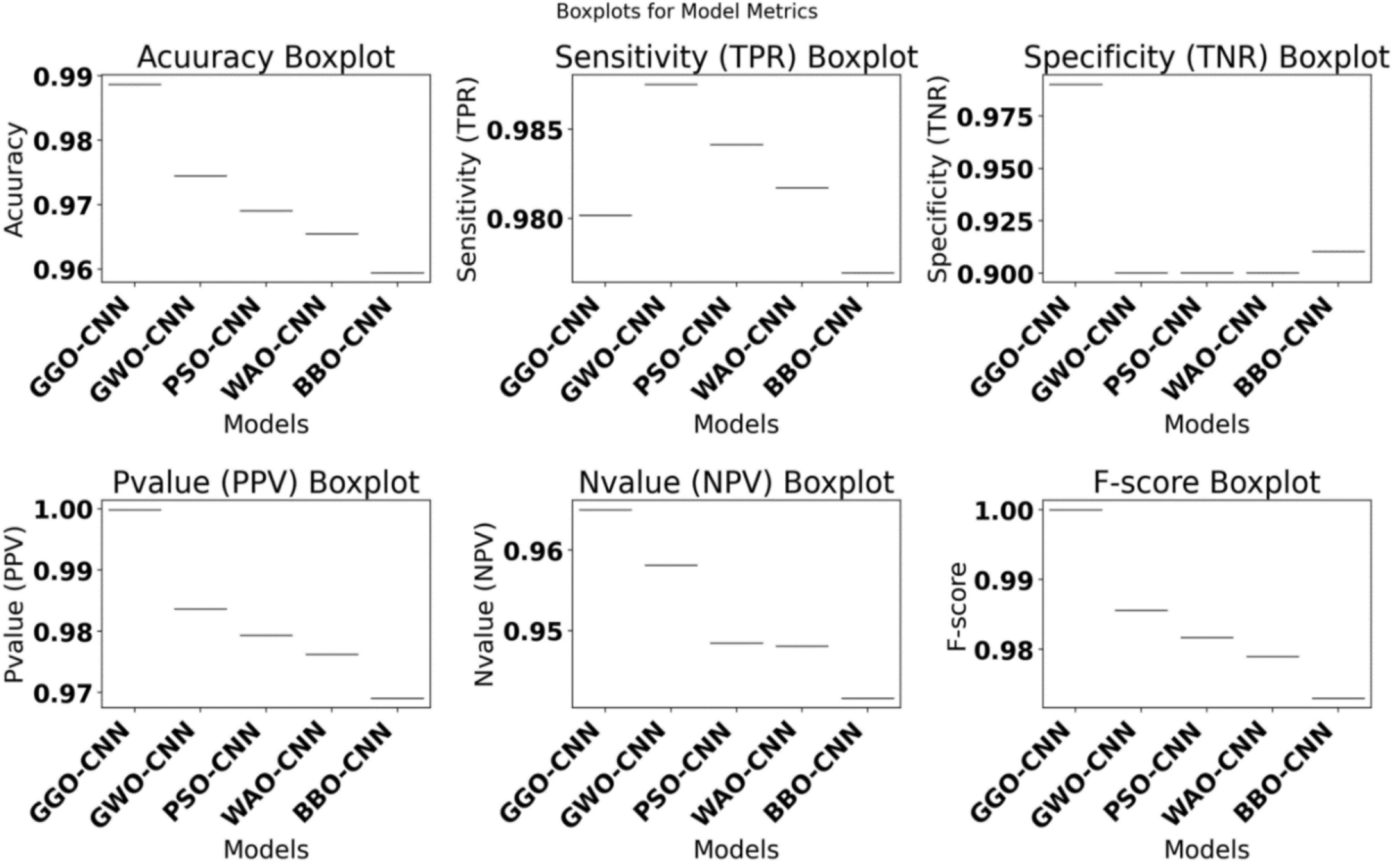
Box plots for model metrics to the suggested and compared algorithms.

**Fig. 9:**
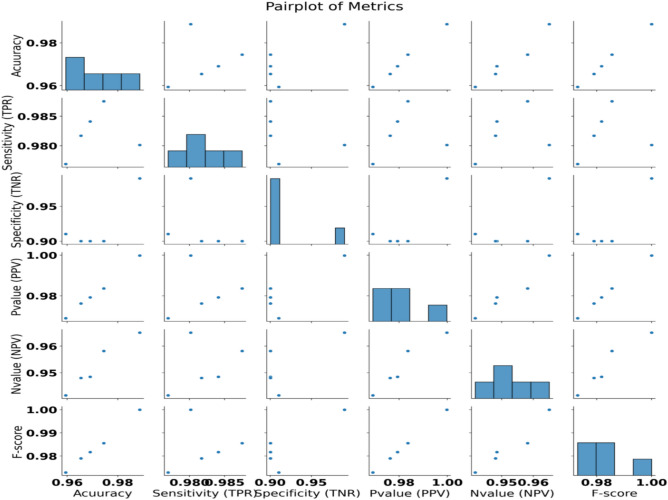
Pair plot of metrics.

The chosen features are fed into the optimized classifiers since the outcomes of applying the suggested feature selection approach are promising. Figure 7 depicts the optimized classifiers-CNN-based model’s outcomes after being fed the desired feature. The attained accuracy is evaluated and displayed within this figure plot. The suggested methodology achieves an accuracy of 98.8692%, which is more accurate than the results of optimizing the CNN utilizing various optimization approaches. Table 11 presents the suggested system’s classification outcomes using K-nearest neighbor (K-NN), a decision tree (DT), Multi-layer Perceptron (MLP) and optimized-convolutional neural network (CNN) model parameters. Figure 8 depicts box plots of model metrics for suggested and contrasted algorithms. Figure 9 depicts a pair plot of metrics.

**Table 11:** The categorization findings for the suggested system using K-nearest neighbor (K-NN), a decision tree (DT), Multi-layer Perceptron (MLP) and parameter optimization for the convolutional neural network (CNN) model.

Signal Classifier	Accuracy	Sensitivity (TRP)	Specificity (TNP)	P-Value (PPV)	N-Value (NPV)	F1-Score
Hyper parameter-CNN	**0.942397937**	**0.960138366**	**0.924980909**	**0.947825484**	**0.943725052**	**0.943941948**
MLP	0.936034846	0.946953932	0.924420909	0.931296387	0.941255926	0.939058814
KNN	0.924681538	0.929587347	0.920310909	0.91122	0.937145926	0.920310909
DT	0.919879909	0.919879909	0.919879909	0.899677889	0.936714926	0.909665404

Table 12 illustrates the ANOVA test findings for the offered bGGO + CNN approach against the comparable procedures. The ANOVA tests confirmed the bGGO + CNN procedure’s efficacy.

**Table 12:** The findings of the ANOVA for the proposed bGGO technique to categorize KOA.

	**SS**	**DF**	**MS**	**F (DFn, DFd)**	**P value**
Treatment (between columns)	0.02406	4	0.006016	F (4, 45) = 146.2	P < 0.0001
Residual (within columns)	0.001852	45	4.12E-05		
Total	0.02591	49			

The suggested technique was compared to recent related works investigations, as seen in Table 13, and it was found to outperform the current studies despite its complex structure and use of multiple approaches.

**Table 13:** Comparisons between the proposed solution in this paper and related works.

References	Dataset	Purpose	Accuracy
^27^	Two datasets: 1. OAI 2. MOST	1. localize knee joint 2. Classify images of the localized knee joint	-Detection: 100% 99.5% -Classification: 60.3%
^28^	OAI	Score the extent of OA in the knee	66.71%
^29^	(OAI)	1.Detect the knee-region 2. Classify the KOA images	88.2%
^32^	N/A	Detect knee joints and classified the detected knee joint images	69.7%
^33^	OAI	Classify the KOA images	71%
^34^	OAI	Classify KOA images	72.7%
^35^	Clinical hospital center of Rijeka	Detect anterior cruciate ligament damage at its early stage	98%
^36^	OAI	Detect KOA at early stage	81.69%
^37^	OAI	Extract the features from OAI dataset and then classify OAI images	71.33%
**Proposed Method**	OAI	Extract the features from OAI dataset, select the best features from them using optimization and then classify images in the dataset	**98.869%**

## Conclusion

A deep learning technique has been presented in this paper to classify knee joint osteoarthritis automatically. KOA categorization was performed using a unique bGGO optimization algorithm based on CNN. The appropriate collection of features is obtained using deep learning and a transfer learning approach. The most prevalent features are taken from the dataset’s photos using various DL pre-trained models, particularly ResNet-50. The collected features were then optimized to minimize their number by Greylag Goose Optimization (bGGO) in binary form to increase accuracy and remove unnecessary features. After applying various classifiers and optimization algorithms to the features that GGO had chosen, classification metrics were computed; the suggested methodology attained an accuracy of 0.988692, a sensitivity of 0.980156, and a specificity of 0.990089. In contrast to similar work, the simulated outcomes outperform those. These findings support the suggested system’s use as an effective diagnostic tool for the early detection of KOA. On the other hand, a statistical analysis was carried out to demonstrate the validity of the suggested framework.

## Figures and Tables

**Fig. 10:**
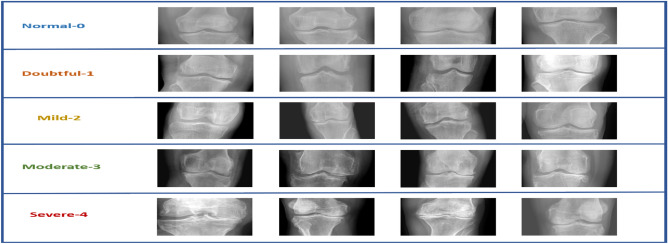
Samples of the utilized datasets.

**Fig. 11:**
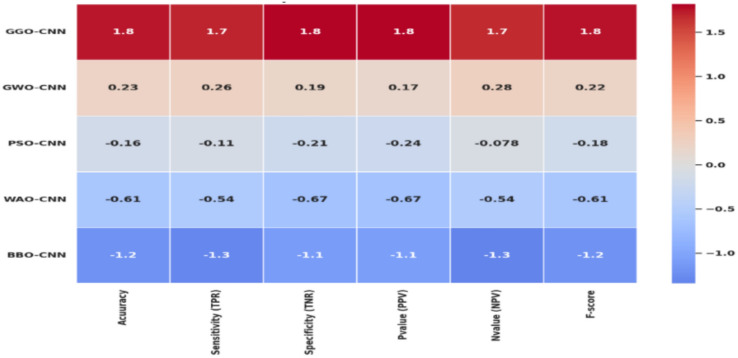
Z-score heatmap of model performance metrices to the GGO-CNN and other comparable approaches.

**Fig. 12:**
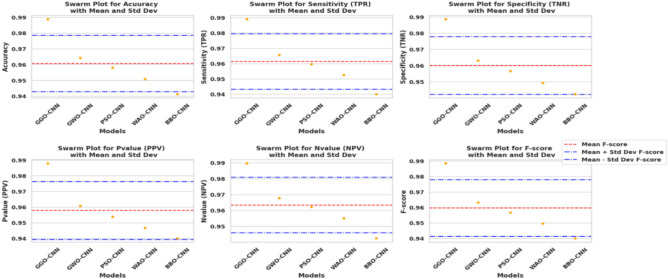
Swarm plots for metrics across model.

**Fig. 13:**
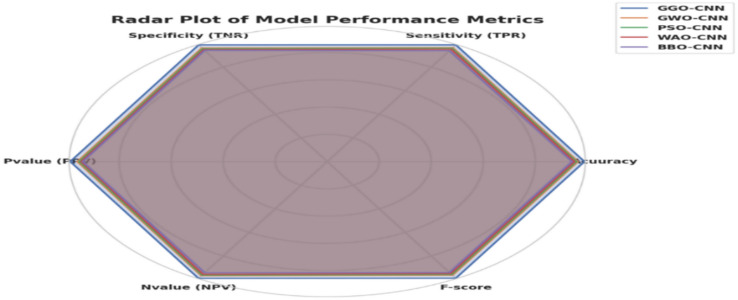
Radar plot of model performance metrics.

**Fig. 14:**
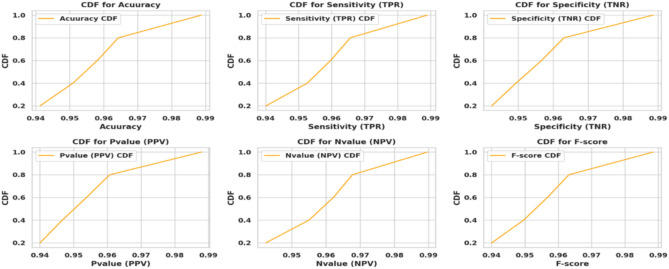
CDF plots for metrics across models**.**

**Fig. 15:**
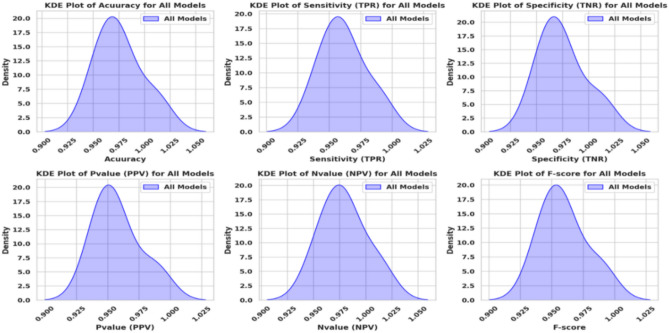
Kernel density estimation plots for model metrics.

**Fig. 16:**
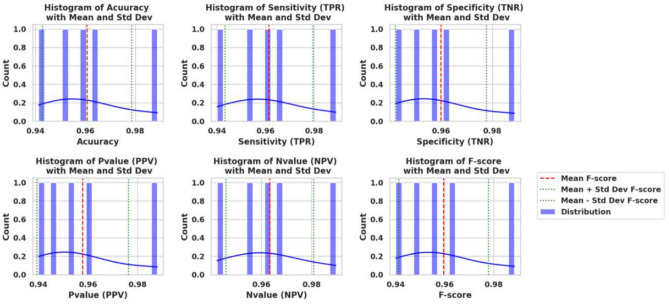
Histograms with mean and standard deviation.

**Fig. 17:**
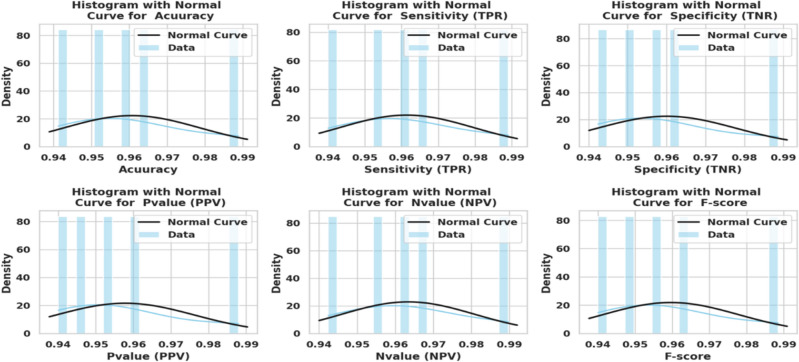
Histograms with normal distribution curve for metrics across models.

**Fig. 18:**
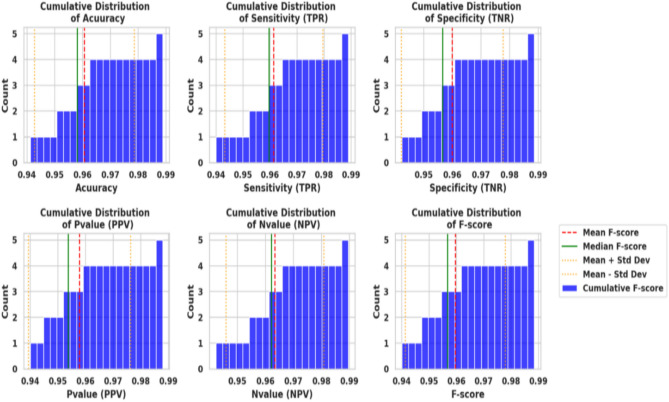
Cumulative distribution plots for metrics across models.

**Fig. 19:**
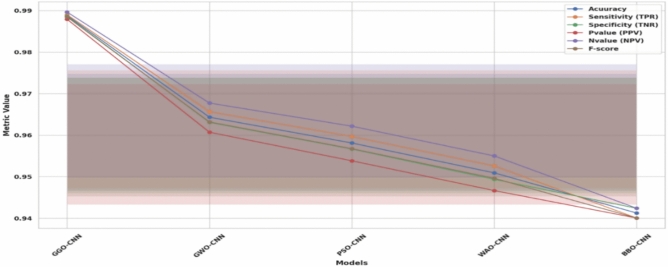
Line plots with confidence intervals for metrics across models.

**Fig. 20:**
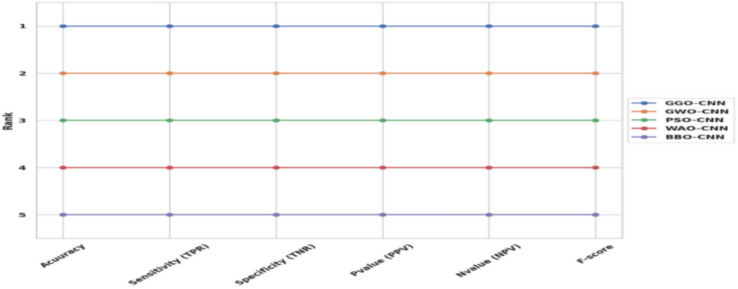
Bump chart of model rankings across different metrices.

**Fig. 21:**
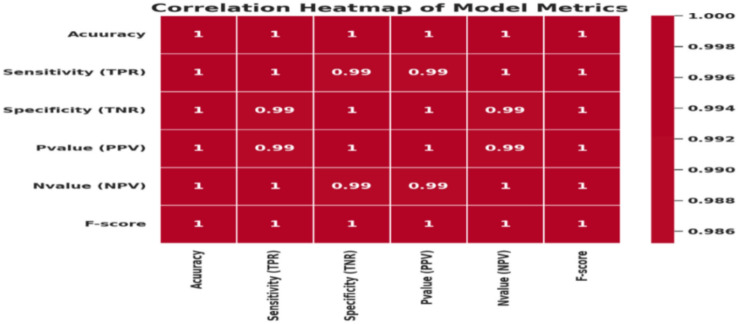
Correlation heatmap of model metrics.

**Fig. 22:**
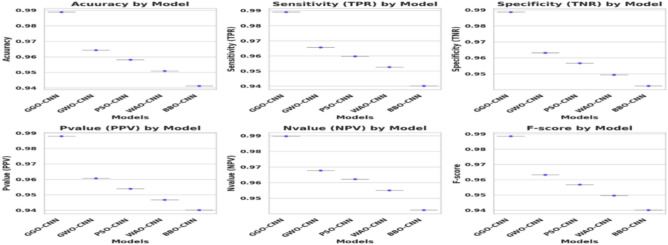
Strip plot with box plot overlay of model metrices.

**Fig. 23:**
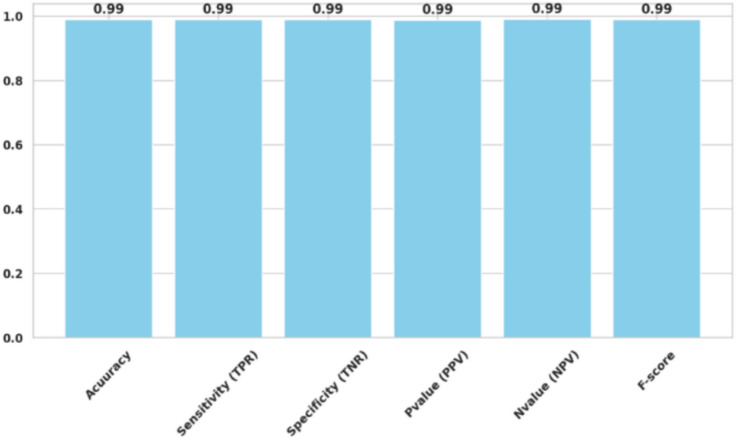
Waterfall chart for model metric breakdown.

**Fig. 24:**
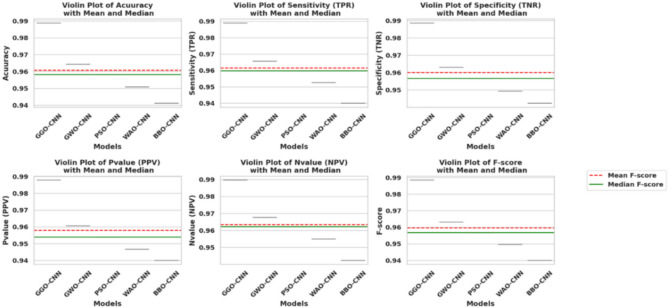
Violin plots with means and median for metrics across models.

**Fig. 25:**
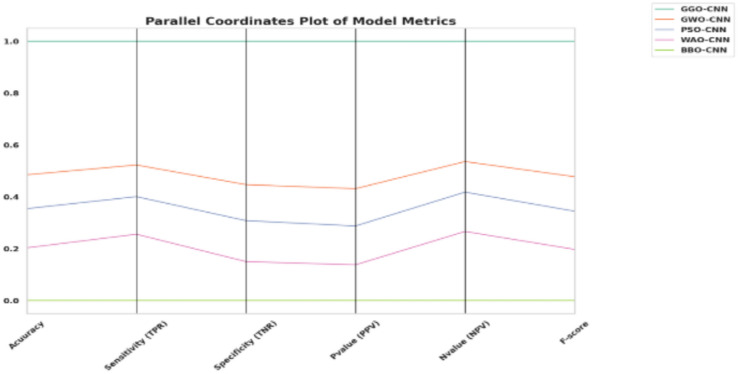
Parallel coordinates plot of model metrics.

**Fig. 26:**
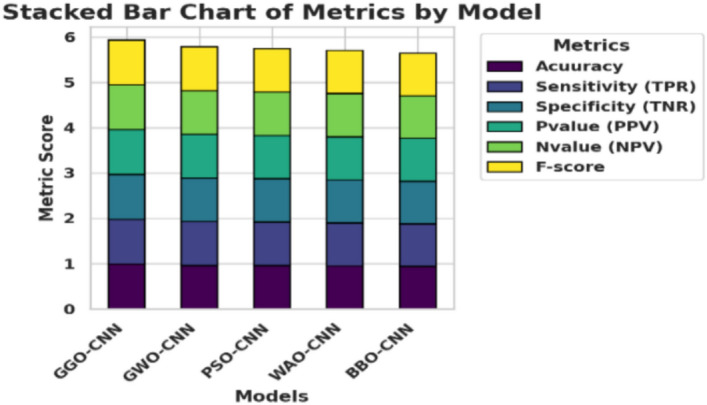
Stacked bar chart of metrics by model.

**Fig. 27:**
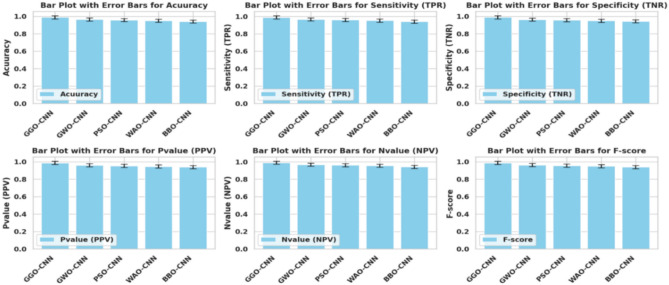
Bar plots with error bars for metrics across models.

**Fig. 28:**
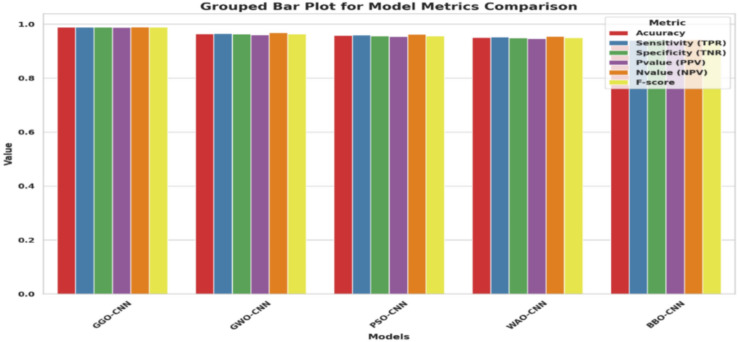
Grouped bar plot for model metrics comparison.

## Data Availability

The data that support the findings of this study are openly available at [https://www.kaggle.com/datasets/farjanakabirsamanta/osteoarthritis-prediction].

## Appendix

The proposed system’s effectiveness was confirmed by testing it on other datasets.

I. Dataset Description.

The Digital Knee X-ray Images dataset (Gornale & Patravali) contains an extensive collection of knee X-ray images^51^. The dataset consists of 1650 digital X-ray pictures of the knee joint obtained from reputable hospitals and diagnostic centers. The X-ray images are captured using a PROTEC PRS 500E X-ray machine. The original photos are 8-bit greyscale images. Each radiographic knee X-ray image is manually annotated/labeled by two medical specialists using KL grades. Figure 10 depicts some samples from the dataset. The KL grading system assigns 5 grades to knee OA severity based on radiographs, with ‘Grade 0’ indicating normal knee and subsequent grades indicating evolution of KOA.

II. Apply the proposed approach.

III. Feature Extraction and Selection Results.

A. Feature Extraction Results.

The extraction method succeeded in identifying the most significant features of the categorization task using four pretrained model (see Table

**Table 14:** Assessing the characteristics that were derived with CNN deep neural networks.

Model	Accuracy	Sensitivity (TRP)	Specificity (TNP)	P-Value (PPV)	N-Value (NPV)	F1-Score
Google-Net	83.6735%	84.2105%	83.1683%	82.4742%	84.8485%	83.3333%
**ResNet-50**	**86%**	**86.3158%**	**85.7143%**	**84.5361%**	**87.3786%**	**85.4167%**
Alex-Net	81.4433%	81.7204%	81.1881%	80%	82.8283%	80.8511%
VGG-19	80.2083%	80.4348%	80%	78.7234%	81.6327%	79.5699%

14).

B. Feature Selection.

The results of the criteria for evaluation rely on the suggested feature selection strategy are shown in Table

**Table 15:** Evaluation of the suggested optimization algorithm against alternative optimization algorithms for the chosen set of features.

Metric	bGGO	bGWO	bPSO	bBA	bGA	bMVO	bSBO	bFA	bWOA
Avg. Error	**0.31641**	0.46721	0.44861	0.34041	0.34891	0.38051	0.39328	0.37178	0.32616
Avg. Select-Size	**0.37471**	0.60511	0.51301	0.63781	0.57051	0.64431	0.69068	0.59338	0.53996
Avg. Fitness	**0.37461**	0.54751	0.50861	0.40471	0.42871	0.43871	0.46558	0.45158	0.38436
Best Fitness	**0.34241**	0.46621	0.41191	0.39441	0.35391	0.38181	0.44728	0.37678	0.51746
Worst Fitness	**0.42861**	0.56381	0.52701	0.48091	0.47191	0.46151	0.43378	0.49478	0.59716
Standard deviation-Fitness	**0.20991**	0.34421	0.30961	0.25571	0.26151	0.27171	0.24858	0.28438	0.22736

^15^, along with a comparison to the other approaches: bGGO, bFA, bSBO, bGWO, bPSO, bBA, bGA, bMVO, and bWOA.

### IV. Classification Results

The suggested methodology achieves an accuracy of 98.8692% (see Table

**Table 16:** The categorization findings for the suggested system utilizing four different classifiers.

Signal Classifier	Accuracy	Sensitivity (TRP)	Specificity (TNP)	P-Value (PPV)	N-Value (NPV)	F1-Score
CNN	0.94556	0.94675	0.94444	0.94118	0.94972	0.94395
MLP	0.92262	0.92593	0.91954	0.91463	0.93023	0.92025
KNN	0.90964	0.9125	0.90698	0.90124	0.91765	0.90683
DT	0.89571	0.89873	0.89286	0.8875	0.90361	0.89308

^16^), which is more accurate than the results of optimizing the CNN utilizing various optimization approaches.

The GGO-CNN model outperformed other cutting-edge classifier models built with the CNN approach (see Table

**Table 17:** The Classification outcomes for various optimization algorithms based on CNN.

Model	Accuracy	Sensitivity (TRP)	Specificity (TNP)	P-Value (PPV)	N-Value (NPV)	F1-Score	Time(s)
GGO-CNN	**0.988811934**	**0.989010989**	**0.988624612**	**0.987925357**	**0.989648033**	**0.988467875**	**23.6841**
GWO-CNN	0.964330153	0.965665236	0.963081862	0.960717336	0.967741935	0.963184932	28.1572
PSO-CNN	0.958121827	0.959684487	0.956663941	0.953832753	0.962171053	0.956749672	30.3117
WOA-CNN	0.950933565	0.952593918	0.949367089	0.946666667	0.955008489	0.949621043	34.3214
BBO-CNN	0.941255007	0.940054496	0.942408377	0.940054496	0.942408377	0.940054496	37.6145

^17^), as evidenced by its accuracy of 0.988811934.

Table

**Table 18:** The findings of the ANOVA for the proposed bGGO technique to categorize KOA.

	**SS**	**DF**	**MS**	**F (DFn, DFd)**	**P value**
Treatment (between columns)	0.02522	3	0.008408	F (3, 36) = 250.5	P < 0.0001
Residual (within columns)	0.001208	36	3.36E-05		
Total	0.02643	39			

^18^ illustrates the ANOVA test findings for the offered bGGO + CNN approach against the comparable procedures. The ANOVA tests confirmed the bGGO + CNN procedure’s efficacy.

Figure 11 depicts a Z-score heatmap of model performance metrics to the GGO-CNN and other comparable approaches. The attained accuracy is evaluated and displayed within this figure plot.

Figure 12 swarm plots for metrics across model metrics to the proposed bGGO + CNN and other comparable algorithms. Swarm plot techniques for ranking each metric category from optimum to worst. Each point represents the average outcome across the six metrics in a particular class.

Figure 13 depicts the radar plot of model performance metrics to the proposed bGGO + CNN and other comparable algorithms. A radar chart is a graphical technique that shows multivariate data as a two-dimensional chart with at least three quantitative parameters depicted on axes starting at the same position.

Figure 14 depicts Cumulative distribution function (CDF) plots for metrics across models.

Figure 15 depicts the KDE plots for model metrics. KDE is a non-parametric technique for estimating the probability density function of an arbitrary variable using kernels as weights. It is a form of kernel smoothing for probability density estimation.

Figure 16 depicts the Histograms with mean and standard deviation. It is employed to provide a summary of data, which is evaluated on an interval scale. It is frequently utilized to depict the data distribution’s main characteristics conveniently.

Figure 17 depicts the histograms with normal distribution curve for the six metrics across models.

V. Statistical Analysis and discussion.

Figure 18 depicts the cumulative distribution plots for metrics across models with mean f-score, median mean f-score, mean + standard deviation, mean-standard deviation, and cumulative f-score. Figure 19 depicts the line plots with confidence intervals for metrics across models.

Figure 20 depicts the bump chart of model rankings across different metrices. Every line within the bump charts represents the variance in ranking for each optimizer. As indicates from the figure that bGGO + CNN outperforms other optimization algorithms.

Figure 21 depicts a correlation heatmap of model metrics. A correlation heatmap is a visual representation of the relationship between all the variables in the dataset.

Figure 22 depicts the strip plot with box plot overlay of model metrices to the proposed bGGO + CNN and other comparable algorithms. A single-axis scatter plot called a “strip plot” is employed to show the distribution metric. Plotting the values as dots across a single axis allows for the overlap of dots with an identical value.

Figure 23 depicts the waterfall chart for model metric breakdown. When items are added or deleted, a waterfall chart displays the ongoing total.

Figure 24 depicts violin plots with means and median for metrics across models. Numerical data distributions for one or more categories are shown in a violin plot.

Figure 25 depicts the parallel coordinates plot of model metrics. Figure 26 Shows the stacked bar chart of metrics by model. Figure 27 Depicts the bar plots with error bars for metrics across models. Figure 28. Shows a comparison to the grouped bar plot for model metrics.
